# Differential impacts of internal and external corporate social responsibility on emotional labor among clinicians in Chinese public hospitals: A cross-sectional study

**DOI:** 10.1097/MD.0000000000043276

**Published:** 2025-07-25

**Authors:** Guoyu Luo, Mohd Anuar Arshad, Xianhang Xu, Mengjiao Zhao

**Affiliations:** a School of Management, Universiti Sains Malaysia, Penang, Malaysia; b School of Management, Chongqing Institute of Engineering, Chongqing, China.

**Keywords:** deep acting, emotional labor, organizational identification, perceived CSR, perceived social support, surface acting

## Abstract

Chinese clinicians face significant emotional labor challenges due to an imbalance in the doctor–patient ratio, long working hours, high regional work stress, and cultural expectations. While research on the relationship between corporate social responsibility (CSR) and emotional labor has grown, the specific impact of internal versus external CSR remains unclear. This study aims to explore the differential effects of internal and external CSR on emotional labor, organizational identification (OI), and perceived social support (PSS) among Chinese clinicians, providing insights into how these factors interact to affect clinicians’ emotional well-being. We surveyed clinicians in grade 3A public hospitals across China. Using PLS-SEM with Smart-PLS4, we analyzed 350 valid responses to examine the relationships between internal and external CSR, emotional labor, OI, and PSS. Our findings confirmed that internal CSR negatively affects surface acting and positively affects deep acting (DA), indicating that organizational support fosters authentic emotional engagement rather than superficial emotional responses. The mediating role of OI was also validated, with internal CSR enhancing clinicians’ identification with the organization, reducing emotional dissonance. However, external CSR did not significantly affect either surface or DA, suggesting that social responsibility outside the organization may not directly alleviate emotional labor. The moderating role of PSS was only partially supported, with social support buffering some of the negative effects of emotional labor but not all. This study is significant for policymakers and healthcare managers, as it highlights the critical role of internal CSR in reducing emotional labor among clinicians. The findings suggest that organizations should focus on internal social responsibility practices (such as fostering a supportive work environment and enhancing OI ) to reduce surface acting and promote DA. By doing so, hospitals can create a more sustainable work environment for clinicians, improving their well-being and reducing burnout.

## 1. Introduction

Corporate social responsibility (CSR) is a critical and extensively discussed subject in academic and global business circles.^[1]^ Associated with societal welfare enhancement, improved financial performance,^[2]^ an excellent corporate reputation,^[3]^ and increased customer commitment.^[4]^ Recently, CSR research has shifted focus from an organizational to a micro-perspective, investigating the psychological impact mechanisms on stakeholders. This shift includes special attention to employees’ perceptions of CSR.^[5]^ A previous meta-analysis synthesized the impact of perceived CSR on employee-related outcomes, including job satisfaction, organizational citizenship behavior, commitment, and innovation.^[6]^ Additionally, scholars have highlighted the importance of identifying mechanisms that connect CSR to desirable employee outcomes, such as behaviors, attitudes, and emotional labor.^[7]^

However, researchers have not adequately explored emotional labor in micro-level CSR studies. Emotional labor is the effort employees make to regulate their emotions according to the expectations of their organizations.^[8]^ Grandey broadens the focus beyond Hochschild (1983) findings by stressing 2 regulating modes within emotional labor: deep acting (DA) and surface acting (SA).^[9]^ Emotional labor is integral to healthcare, facilitating patient connections, especially during stressful or painful procedures. It plays a significant role in ensuring patient comfort and safety.^[10,11]^ Some studies have shown that emotional labor results in detrimental psychological effects on workers, including mental health, depression, burnout, turnover, and emotional exhaustion.^[12–14]^ Prior studies on emotional labor have primarily emphasized the individual level,^[15,16]^ resulting in limited knowledge regarding its relationship with organizational variables.^[17]^ While there has been growing interest in the relationship between CSR and emotional labor, most existing research has focused primarily on the impact of external CSR on employees in service industries, with limited attention given to internal CSR practices and their potential influence on emotional labor. Furthermore, research into the predictors of emotional labor and the different mechanisms underlying these relationships is necessary to grasp employees’ psychological and behavioral requirements. This study aims to fill these gaps by exploring and comparing the effects of both internal and external CSR on emotional labor among clinicians in Chinese public hospitals. This context has received limited attention in the literature.

**Figure 1.**
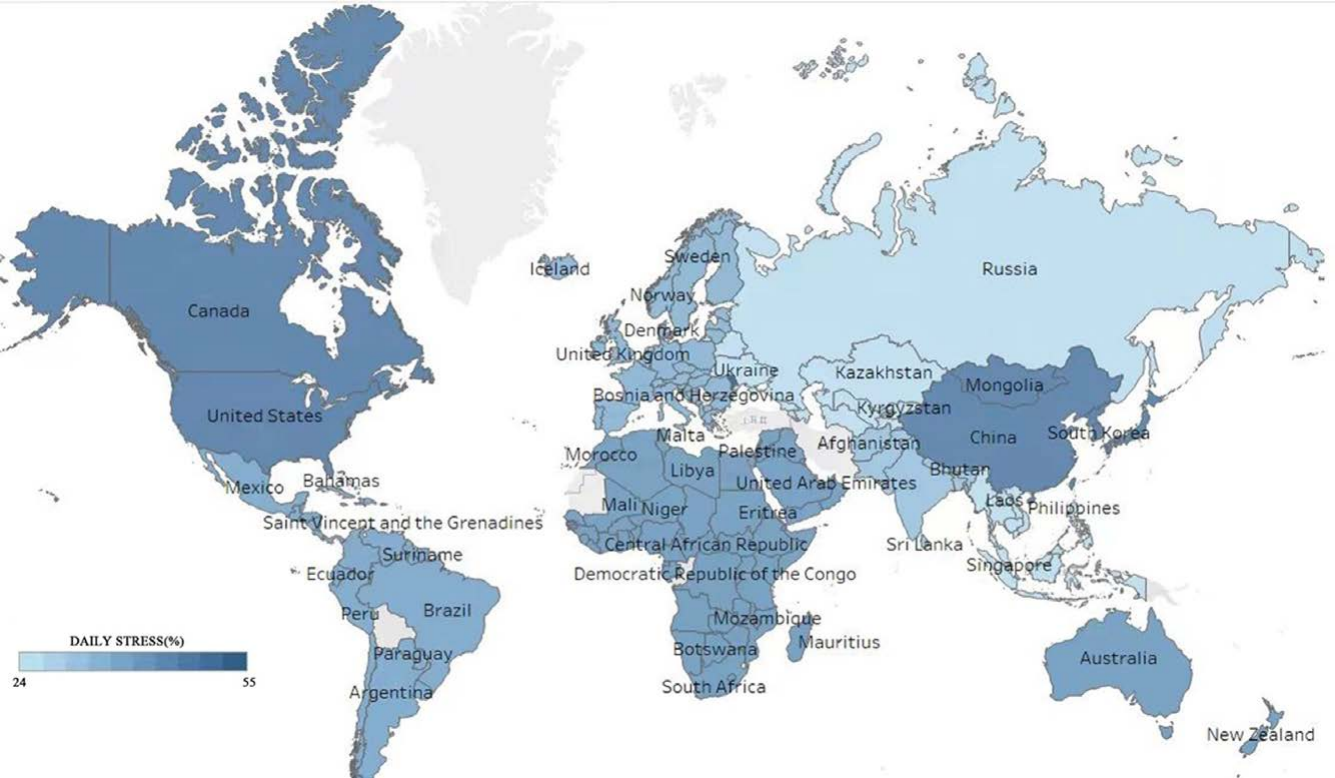
Map of World Daily Stress Index. World map illustrating the Daily Stress Index by country, based on data from Gallup.^[18]^ The map shows the percentage of daily stress experienced by individuals in various countries, with color gradients indicating stress levels. Darker shades represent higher stress levels, ranging from 24% to 55%. East Asia has the highest stress levels worldwide, tied with the United States and Canada, with China leading at a 55% stress level. Source: Author's own creation.

Moreover, exploring emotional labor is particularly critical in East Asia, a region identified by Gallup “Global Workplace Report”^[18]^ as having the highest stress levels worldwide, tied to the United States and Canada, with China leading at a 55% stress level (see Fig. 1). The collectivist culture is further exacerbated in this,^[19]^ emphasizing saving face and high expectations of professional dedication, significantly influencing individuals’ emotional display rules.^[20]^ China’s healthcare sector exemplifies these challenges. According to the 2023 Euro RSCG data,^[21]^ China has a doctor–patient ratio of 1:950, which is significantly lower than the global average of 1:700 and even further below Cuba’s leading ratio of 1:170, indicating an immense workload for doctors who serve approximately 1000 people each. Findings from the Chinese Medical Doctor Association (2022)^[22]^ revealed that 77.8% of doctors worked approximately 12 hours daily, 20% worked between 15 and 18 hours, and 49.2% had at least 2-night shifts per week, with all having experience working continuously for over 24 hours. Coupled with a severe shortage of doctors, prolonged exposure to patients with high levels of negative emotions and physical suffering, and complex doctor–patient relationships, doctors in China face intense emotional labor demands. Additionally, the societal expectation for doctors to uphold medical expertise and moral integrity is intensified by a collectivist culture that prioritizes social harmony and responsibility. These factors make Chinese clinicians particularly vulnerable to the challenges of emotional labor, making it crucial to understand how CSR practices in the Chinese context might alleviate or exacerbate these challenges. Therefore, this study focuses on Chinese clinical doctors in the healthcare industry.

This study aimed to investigate how perceived CSR influences the emotional labor of Chinese clinicians, focusing on the dual mechanisms of organizational identification (OI) and perceived social support (PSS), and provides new perspectives to understand the impact of CSR on emotional labor. Given the inconsistent conclusions of current studies, this study will provide new data and analyses for academics to promote further discussion and research. This study expands the application of CSR in the healthcare industry by suggesting effective CSR strategies and employee support programs for hospital management to enhance physician satisfaction and emotional labor management, thereby improving the quality of healthcare services. This study employed the Perceived CSR Scale, distinct from earlier versions, and marks its initial implementation in the medical domain to investigate interdisciplinary research. Perceived social support is introduced as a moderating variable. Section 2 reviews the literature, section 3 describes the research method, section 4 presents the data analysis and results, section 5 discusses the findings, and section 6 provides the conclusion.

## 2. Literature review and hypotheses

### 2.1. Perceived CSR and emotional labor

CSR is the voluntary action taken by a company to protect the interests of its stakeholders while ensuring long-term financial sustainability.^[23]^ Employees are critical stakeholders of CSR actions. Some scholars have shown that perceived CSR is described as how employees view their organization’s overall CSR activities, and it is based on subjective assessment.^[24,25]^ Several studies indicate that when employees view their organization as actively participating in social responsibility, this perception can deepen employees’ identification with their organization,^[26]^ thereby affecting their attitudes and behaviors,^[27]^ including the choice of emotional labor strategies.^[28]^ Emotional labor strategies include SA and DA.^[9]^ On the one hand, when employees perceive that their company is fulfilling its social responsibilities, this may improve their sense of identification and satisfaction with the organization, leading them to engage in DA as part of their emotional labor because they genuinely identify with and support the company’s values and objectives.^[7]^ Additionally, if employees perceive that the company’s CSR performance is lacking, this could lead to distrust and dissatisfaction with the company, making them more likely to adopt surface-acting strategies in their emotional labor to fulfill the emotional demands of their job despite not truly identifying with them.^[29]^ Consequently, the following hypothesis is formulated:

*H1*: *perceived internal CSR (PICSR) is significantly negatively related to SA.*

*H2: PICSR is significantly positively related to DA.*

*H3: perceived external CSR (PECSR) is significantly negatively related to SA.*

*H4: PECSR is significantly positively related to DA.*

### 2.2. Mediating effect of organizational identification

When an employee feels belonging to an organization and defines their identity based on group qualities, this is called OI.^[30]^ OI positively affects key employee outcomes, including job satisfaction,^[31]^ work engagement,^[32]^ and job performance,^[33]^ organizational commitment.^[34]^ Previous research indicates that CSR activity enhances employees’ OI, subsequently affecting certain employees’ outcomes.^[35,36]^ For example, researchers have discovered that CSR initiatives directed toward internal employees promote perceived respect (employees’ perceptions of how the company regards them), strengthening OI among members.^[37]^ Research by O. Farooq et al revealed that perceived external CSR enhances OI and employee loyalty through improved external reputation.^[38]^ Based on this, we propose the following hypothesis:

*H5: PICSR is significantly positively related to OI.*

*H6: PECSR is significantly positively related to OI.*

Social identity theory posits that a solid organizational identity leads employees to align their personal goals with those of the organization, thus motivating behaviors that benefit the organization.^[39]^ Practices such as organizational citizenship^[40]^ and customer-oriented behavior^[41]^ are good examples of this, significant in the healthcare industry. Therefore, it can be expected that in the context of healthcare service encounters, medical personnel with strong OI will internalize the display rules of the healthcare institution, engaging in DA.^[42]^ Mishra research supports this viewpoint by revealing that employees with lower OI are more likely to exhibit SA to mask their genuine feelings.^[42]^ Based on the preceding discussion, we propose the following hypotheses:

*H7: OI is significantly negatively related to SA.*

*H8: OI is significantly positively related to DA.*

Studies conducted by Khan and other scholars confirm that employees, when perceiving internal CSR within a company, increase their sense of organizational support, thereby reducing surface-level performance in the workplace.^[43]^ Scholars have shown that the perception of internal CSR also actively reinforces employees’ identification with the organization and prompts them to exhibit more deep-acting behaviors.^[7]^ Cheng et al revealed that impression management motives and organizational identity partially mediate the effect of perceived external prestige on SA.^[44]^ Deniz found that employees’ positive perceptions of the organization’s external reputation are associated with OI, leading employees to be more inclined to engage in emotional effort for the organization.^[45]^ This suggests that higher levels of OI increase the connection between organizational objectives and self-goals, aligning organizational actions with personal interests. Consequently, employees might internalize and display the emotions expected by the organization during interactions with clients,^[35,36]^ known as DA. Based on the preceding discussion, we propose the following hypothesis:

*H9: OI will significantly mediate the effect of PICSR and SA.*

*H10: OI will significantly mediate the effect of PICSR and DA.*

*H11: OI will significantly mediate the effect of PECSR and SA.*

*H12: OI will significantly mediate the effect of PECSR and DA.*

### 2.3. Moderating effect of perceived social support

Medical work is characterized by high risk and workload, and social support is especially critical when healthcare workers are under pressure. Perceived social support (PSS) is the extent to which employees believe they can obtain material or emotional support from family, friends, and other significant interpersonal relationships.^[46]^ Research indicates that the sufficiency of social support is directly linked to the intensity of reported psychological and physical symptoms.^[47]^ Alternatively, it serves as a buffer in dealing with stressful life events and associated symptoms.^[48]^ When healthcare workers feel support from their families and colleagues, they will be more willing to show positive emotions at work, even in the face of challenges and pressures, which will help them recover from emotional labor, thus reducing the depletion and potential negative effects of emotional labor.^[49]^ Moreover, PSS can improve employees’ job satisfaction,^[50]^ and high job satisfaction motivates employees to express genuine emotions rather than masking their true feelings to meet organizational expectations.^[51]^ Hence, this study puts forth the following hypothesis:

*H13: PSS will significantly moderate the relationship between PICSR and SA.*

*H14: PSS will significantly moderate the relationship between PICSR and DA.*

*H15*: *PSS will significantly moderate the relationship between PECSR and SA.*

*H16: PSS will significantly moderate the relationship between PECSR and DA.*

**Figure 2.**
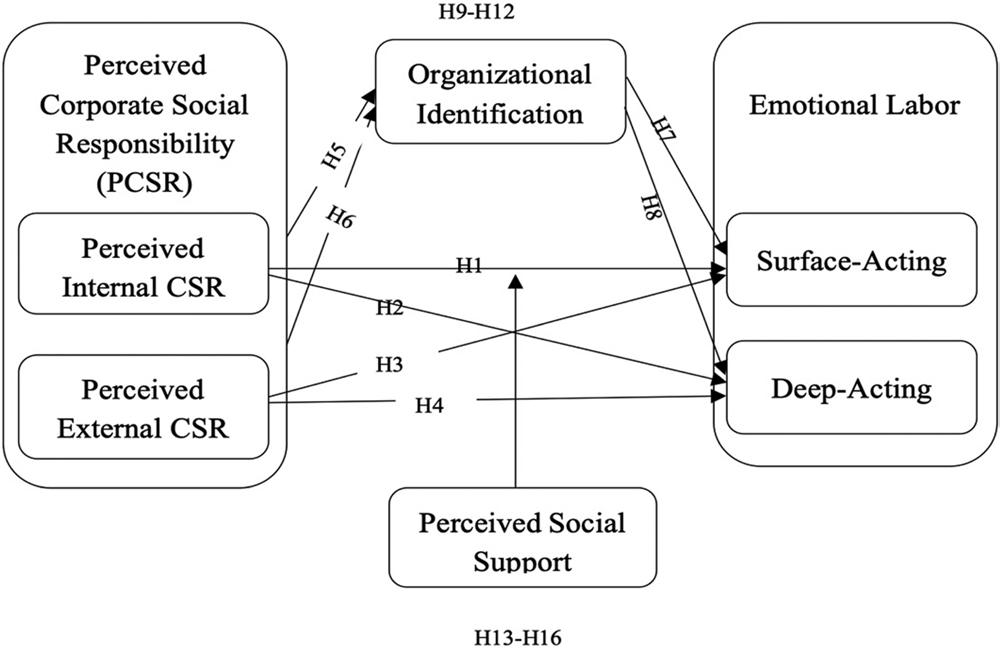
Theoretical framework. This figure illustrates the relationships between the key constructs in the study. Perceived corporate social responsibility (PCSR), with its dimensions of perceived internal CSR and perceived external CSR, influences organizational identification, affecting emotional labor. Emotional labor is represented by 2 forms: surface acting and deep acting. Additionally, perceived social support is a moderating variable that impacts the relationships among PCSR, organizational identification, and emotional labor.

Based on the above theoretical assumptions, we constructed a model depicting the relationship between PCSR, EL, OL, and PSS (Fig. 2).

## 3. Method

### 3.1. Sampling

This study employs purposive sampling based on the specificity of the study population and the strong relevance of emotional labor to the research. Firstly, the study focuses on clinicians in Grade 3A public hospitals in China. Random sampling might include doctors with lower levels of emotional labor, which could affect the study’s accuracy. Therefore, the study selected the top 4 general hospitals and 3 representative specialty hospitals from the 22 Grade 3A public hospitals in Taiyuan, Shanxi Province (First Hospital of Shanxi Medical University, Second Hospital of Shanxi Medical University, Shanxi Baiqiu’en Hospital, Shanxi Provincial People’s Hospital, Shanxi Cardiovascular Hospital, Children’s Hospital of Shanxi, and Shanxi Provincial Hospital of Traditional Chinese Medicine). These hospitals generally experience high patient volumes and greater medical service demands. Hence, doctors will likely face more intense emotional labor challenges, making them more representative of the research needs. Secondly, the study focuses on 5 key departments: emergency, cardiology, pediatrics, obstetrics and gynecology, and intensive care. According to Medscape’s “2023 Physician Burnout and Depression Report,”^[52]^ clinicians in these departments have been found to experience the highest levels of burnout and depression, indicating that they are likely to face more significant pressures and emotional labor challenges, thus providing high-quality data to support the research.

Several measures were implemented to minimize the selection bias potentially introduced by purposive sampling. First, clear inclusion criteria were established, and only clinicians from Grade 3A public hospitals were selected to ensure comparability among the participants. Second, a combination of general and specialized hospitals, along with various departments, was included to enhance the diversity of the sample. In addition, both survey questionnaires and interviews were utilized, and existing research data were referenced for validation to ensure the representativeness of the selected sample. Finally, in discussing the research findings, careful attention will be given to the applicability of the sample, avoiding overgeneralization of the conclusions.

A total of 482 questionnaires were distributed for this study. Strict exclusion criteria were applied to ensure data quality and reliability. Participants with less than 1 year of work experience were excluded, as were those under 25. Additionally, questionnaires demonstrating identical responses across all items or showing inconsistencies and lack of logical coherence were excluded from the final analysis. After eliminating invalid questionnaires, 350 valid responses were obtained, yielding an effective response rate of 72.61%. Among the sample, 85.14% were female. Most respondents were 36 to 45 years old, accounting for 52.86%. Respondents with a master’s degree or higher comprised 89.14% of the sample. Regarding work experience, 66.29% had more than 10 years of experience. The highest proportion of monthly income fell within the 7000 to 9000-yuan range at 44.86% (see Table 1).

**Table 1:** Respondent demographics (N = 350).

Demographics		Frequency	Percentage (%)
Gender	Male	52	14.86%
	Female	298	85.14%
Age group	25 yr old and below	0	0%
	26–35 yr	55	15.71%
	36–45 yr	185	52.86%
	46–55 yr	86	24.57%
	Above 55 yr	24	6.86%
Highest level of education	Bachelor’s degree	28	8%
	Master’s degree	275	78.57%
	Doctor of Philosophy	37	10.57%
	Others	10	2.86%
Years of service as a permanent employee	1–5 yr	32	9.14%
	6–10 yr	86	24.57%
	More than 10 yr	232	66.29%
Marital status	Single	46	13.14%
	Married	293	83.71%
	Divorced	10	2.86%
	Widow	1	0.29%
Monthly income (after tax)	3000 yuan and below	13	3.71%
	Between 3000 and 5000 yuan	23	6.57%
	Between 5000 and 7000 yuan	96	27.43%
	Between 7000 and 9000 yuan	157	44.86%
	Above 9000 yuan	61	17.43%
Total		350	100%

### 3.2. Measures

We utilized the conceptual definitions of model constructs (variables) from the existing literature^[9,53–55]^ and the context of this study to present the operational definitions of the 6 constructs in Table 2 using question items as observation sets.

**Table 2:** Operational definitions.

Variables	Operational definitions	Reference sources
Deep acting (DA)	Adjusting internal emotions to align with desired ones.	Grandey^[9]^
Surface-acting (SA)	Displaying emotions without altering the underlying ones.	Grandey^[9]^
Perceived external CSR (PECSR)	Initiatives aimed at promoting stewardship of the local community, natural environment, or consumers.	El Akremi et al^[53]^
Perceived internal CSR (PICSR)	Stewardship practices specifically target the internal workforce.	El Akremi et al^[53]^
Perceived social support (PSS)	The individual’s perception of accessing external support.	Sarason^[54]^
Organizational identification (OI)	One particular type of social identification involves feeling a sense of unity or belonging to the organization.	Ashforth and Mael^[55]^

The PICSR, PECSR, and PSS scales in this study were measured using a seven-point Likert scale, ranging from 1 (“strongly disagree”) to 7 (“strongly agree”). Other scales, such as OI, SA, and DA, were measured using a five-point Likert scale, ranging from 1 (“completely disagree”) to 5 (“completely agree”).

OI. This is a six-item scale for assessing OI.^[55]^ The Cronbach alpha was 0.929.

PICSR. Six items were adapted from Farooq et al^[56]^ to measure the PICSR. The Cronbach alpha was 0.925.

PECSR. This is a ten-item scale that measures PECSR,^[56]^ which includes 3 dimensions: environment oriented CSR, community oriented CSR, and consumer oriented CSR. The Cronbach alpha was 0.896.

DA. This study used the Emotional Labor Scale designed by Diefendorff,^[57]^ which consists of 4 items related to DA. Cronbach alpha was 0.893.

SA. This study used Diefendorff Emotional Labor Scale,^[57]^which consists of 7 SA items. Cronbach alpha was 0.917.

PSS. This is a twelve-item scale for assessing PSS.^[46]^ Cronbach alpha was 0.954.

## 4. Data analysis and results

### 4.1. Reliability and validity

In this study, we conducted a confirmatory factor analysis using Smart PLS 4.0 to test the reliability and validity of the measurements. As shown in Table 3, Cronbach alpha and composite reliability for all constructs exceeded 0.70, confirming the reliability of the constructs.^[58]^ Moreover, the average variance extracted (AVE) values exceeded the threshold 0.5,^[58]^ which indicates that the measurement tool used in this study had sufficient convergent validity.

**Table 3:** Reliability and convergent validity of each construct.

Second-order variable	First-order variable	Item	Factor loading	Cronbach alpha	CR	AVE
	Deep acting	DA1	0.88	0.893	0.926	0.758
		DA2	0.839			
		DA3	0.917			
		DA4	0.846			
	Surface-acting	SA1	0.786	0.917	0.934	0.669
		SA2	0.792			
		SA3	0.852			
		SA4	0.792			
		SA5	0.781			
		SA6	0.852			
		SA7	0.866			
PECSR				0.896	0.87	0.69
	Environment oriented CSR	PECSR11	0.835	0.851	0.9	0.691
		PECSR12	0.81			
		PECSR13	0.847			
		PECSR14	0.833			
	Community oriented CSR	PECSR21	0.875	0.849	0.908	0.768
		PECSR22	0.865			
		PECSR23	0.888			
	Consumer oriented CSR	PECSR31	0.88	0.874	0.923	0.799
		PECSR32	0.91			
		PECSR33	0.893			
	Perceived internal CSR	PICSR1	0.789	0.925	0.942	0.73
		PICSR2	0.9			
		PICSR3	0.829			
		PICSR4	0.851			
		PICSR5	0.848			
		PICSR6	0.903			
	Organizational identification	OI1	0.871	0.929	0.944	0.739
		OI2	0.856			
		OI3	0.844			
		OI4	0.854			
		OI5	0.872			
		OI6	0.861			
	Perceived social support	PSS1	0.849	0.954	0.96	0.666
		PSS2	0.846			
		PSS3	0.842			
		PSS4	0.799			
		PSS5	0.837			
		PSS6	0.809			
		PSS7	0.784			
		PSS8	0.795			
		PSS9	0.828			
		PSS10	0.835			
		PSS11	0.751			
		PSS12	0.812			

AVE = average variance extracted, CR = composite reliability.

Finally, discriminant validity was assessed by comparing the square root of each construct’s AVE with its Pearson correlation coefficients with other constructs, following the Fornell–Larcker criterion.^[59,60]^ When the square root of a construct’s AVE exceeds its correlation coefficient with any other construct, it verifies discriminant validity.^[61]^ Table 4 shows that for the diagonal constructs in this investigation, the square root of the AVE was more significant than the off-diagonal correlation coefficient. As a result, most constructs demonstrated strong discriminant validity.

**Table 4:** Discriminant validity (Fornell–Larcker criterion).

	1	2	3	4	5	6	7	8
1. Deep acting	**0.871**							
2. Surface-acting	-0.626	**0.818**						
3. Community oriented CSR	0.287	-0.271	**0.876**					
4. Consumer oriented CSR	0.279	-0.313	0.529	**0.894**				
5. Environment oriented CSR	0.278	-0.306	0.545	0.537	**0.831**			
6. Perception internal CSR	0.418	-0.551	0.4	0.402	0.521	**0.854**		
7. Organizational identification	0.485	-0.567	0.38	0.415	0.528	0.607	**0.86**	
8. Perceived social support	0.3	-0.443	0.323	0.371	0.356	0.469	0.331	**0.816**

### 4.2. Common method bias

We used Harman single-factor test to assess common method bias. The results indicated that 8 factors with eigenvalues exceeding one accounted for 72.161% of the total variance. The first principal factor explained 36.652% of the variance, below the critical threshold of 40%. Consequently, this study showed no significant evidence of common method bias.

### 4.3. Model hypothesis testing

We used partial least squares analysis to assess the hypotheses of this study. In partial least squares, the internal model consists of path relationships between structures. The *P*-values and path coefficients were estimated using this model. Path coefficients show the causal relationship between the observable and latent variables by indicating the direction and degree of association between the variables. The *R*² value represents the percentage of variance in the dependent variable explained by the model, which means its predictive ability.

#### 4.3.1. Testing the direct effect

**Figure 3.**
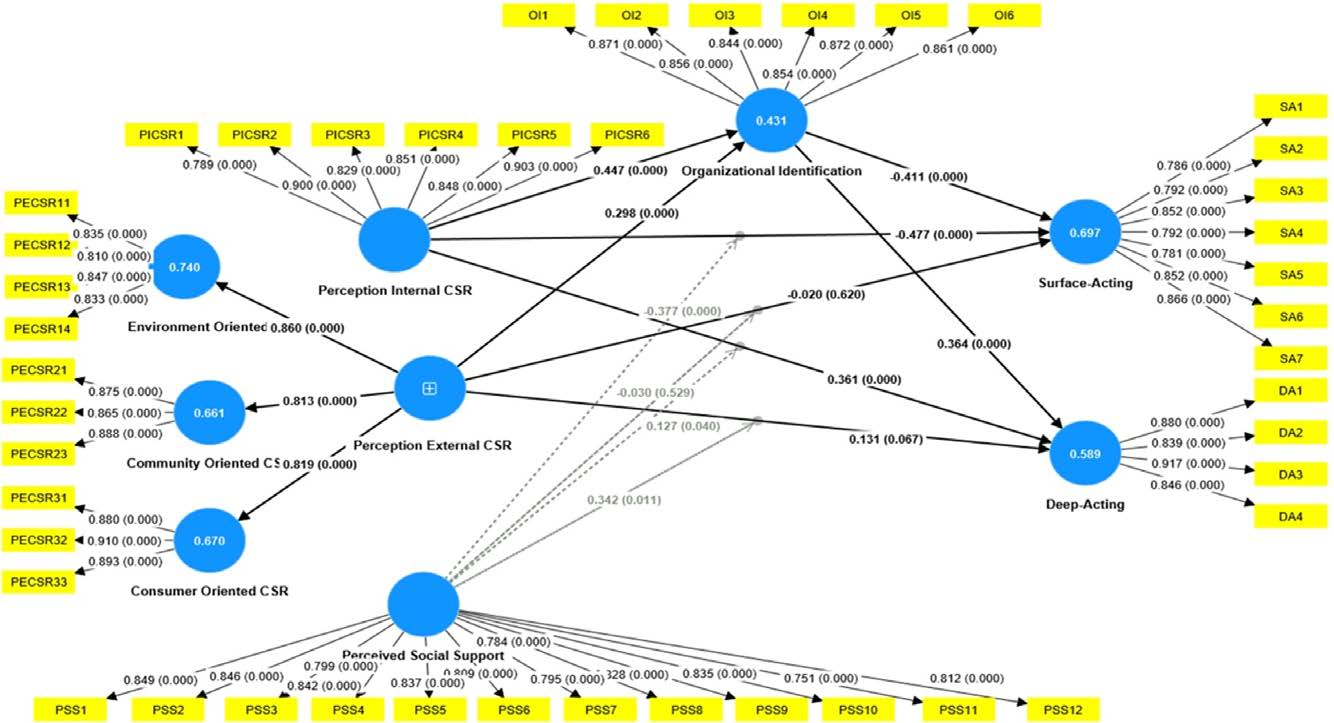
Standardized path coefficients and significance. Yellow boxes represent measurement indicators (e.g., PICSR1–PICSR6), and blue circles represent latent variables (e.g., PICSR). The numbers on the arrows, such as “perception internal CSR → PICSR1 0.789 (0.000),” show the factor loadings (0.789), indicating a strong explanatory power of the indicator for the latent variable. The value in parentheses (.000) is the *P*-value, indicating statistical significance (*P* < .05). The relationships between independent variables (PECSR, PICSR), mediator (OI), moderator (PSS), and dependent variables (SA, DA) are shown by the arrows. For example, “PICSR → OI 0.447 (0.000)” represents the path coefficient (0.447), indicating the strength of the relationship. Values close to + 1 indicate a strong positive correlation, and values close to −1 indicate a strong negative correlation. The *P*-value (.000) indicates statistical significance.

From Table 5 and Figure 3, it can be seen that PICSR is significantly negatively related to SA, supporting H1 (β = −0.477, *P* < .01); PICSR has a significant positive effect on DA, supporting H2 (β = 0.361, *P* < .01); PECSR does not have a significant negative impact on SA, not supporting H3 (β = −0.020, *P* > .05); PECSR does not have a significant positive effect on DA, not supporting H4 (β = 0.131, *P* > .05); PICSR significantly and positively impacts OI, supporting H5 (β = 0.447, *P* < .01); PECSR significantly and positively impacts OI, supporting H6 (β = 0.298, *P* < .01); OI has a significant negative effect on SA, supporting H7 (β = −0.411, *P* < .01); OI has a considerable positive impact on DA, supporting H8 (β = 0.364, *P* < .01).

**Table 5:** Hypothesis testing for direct effect.

No.	Path	β	STDEV	t	*P*	*R* ^2^	Decision
H1	PICSR ≥ SA	-0.477	0.049	9.830	.000	0.697	Supported
H2	PICSR ≥ DA	0.361	0.064	5.643	.000	0.589	Supported
H3	PECSR ≥ SA	-0.020	0.041	0.495	.620	0.697	Not Supported
H4	PECSR ≥ DA	0.131	0.071	1.833	.067	0.589	Not Supported
H5	PICSR ≥ OI	0.447	0.052	8.557	.000	0.431	Supported
H6	PECSR ≥ OI	0.298	0.053	5.640	.000	0.431	Supported
H7	OI ≥ SA	-0.411	0.027	15.236	.000	0.697	Supported
H8	OI ≥ DA	0.364	0.046	7.990	.000	0.589	Supported

DA = deep acting, OI = organizational identification, PECSR = perceived external corporate social responsibility, PICSR = perceived internal corporate social responsibility, PSS = perceived social support, SA = surface acting.

#### 4.3.2. Testing the mediating effect

Furthermore, this study examined the mediating influence of OI (see Table 6). The findings show that OI substantially mediates the relationships between PICSR, PECSR, SA, and DA (95% confidence intervals exclude zero, *P* < .01). Therefore, hypotheses H9, H10, H11, and H12 were supported.

**Table 6:** Hypothesis testing for mediating effect.

No.	Relationship	Effect	T	*P*	95% lower	95% upper	Decision
H9	PICSR ≥ OI ≥ SA	-0.184	7.412	.000	-0.236	-0.138	Supported
H10	PICSR ≥ OI ≥ DA	0.163	5.240	.000	0.109	0.232	Supported
H11	PECSR ≥ OI ≥ SA	-0.123	5.136	.000	-0.171	-0.077	Supported
H12	PECSR ≥ OI ≥ DA	0.109	5.203	.000	0.070	0.152	Supported

DA = deep acting, OI = organizational identification, PECSR = perceived external corporate social responsibility, PICSR = perceived internal corporate social responsibility, PSS = perceived social support, SA = surface acting.

* *P* < .05 (based on a two-tailed test with 5000 bootstrapping).

#### 4.3.3. Testing the moderating effect

We further tested the moderating effect of PSS. The results (Table 7) suggest that PSS moderated the impact of PICSR on SA (β = −0.377, SE = 0.050, t = 7.471, *P* < .01), confirming Hypothesis 13. Similarly, we found that PSS moderated the effect of PICSR on DA (β = 0.127, SE = 0.062, t = 2.055, *P* < .05), thus supporting Hypothesis 14. However, the moderating role of PSS between PECSR and SA is not supported (*P* > .05); therefore, Hypothesis 15 is not confirmed. Finally, PSS moderated the effect of PECSR on DA (β = 0.342, SE = 0.134, t = 2.551, *P* < .05), supporting Hypothesis H16. To further validate the significant moderating effects of PSS, we conducted a simple slope analysis for the supported hypotheses (H13, H14, and H16).

**Table 7:** Hypothesis testing for moderating effect.

No.	Path	β	STDEV	*t*	*P*	*R* ^2^	Decision
H13	PSS*PICSR ≥ SA	-0.377	0.050	7.471	.000	0.697	Supported
H14	PSS*PICSR ≥ DA	0.127	0.062	2.055	.040	0.589	Supported
H15	PSS*PECSR ≥ SA	-0.030	0.048	0.629	.529	0.697	Not supported
H16	PSS*PECSR ≥ DA	0.342	0.134	2.551	.011	0.589	Supported

DA = deep acting, PECSR = perceived external corporate social responsibility, PICSR = perceived internal corporate social responsibility, PSS = perceived social support, SA = surface acting.

* *P* < .05 (based on a two-tailed test with 5000 bootstrapping).

##### 4.3.3.1. Simple slope analysis of the moderating effect of PSS on the relationship between PICSR and SA

In this study, the independent variable (PICSR) and the moderating variable (PSS), with the dependent variable (SA), were subjected to a simple slope analysis, the results of which are presented in Table 8 as follows.

**Table 8:** Regression coefficients of PICSR on SA at different PSS levels.

Moderator level	Regression coefficient	Standard error	*t*	*P*	95% CI	
Mean	-0.227	0.029	-7.957	.000	-0.283	-0.171
High level (+1 SD)	-0.363	0.040	-8.992	.000	-0.442	-0.284
Low level (-1 SD)	-0.090	0.028	-3.265	.001	-0.145	-0.036

PICSR = perceived internal corporate social responsibility, PSS = perceived social support, SA = surface acting.

**Figure 4.**
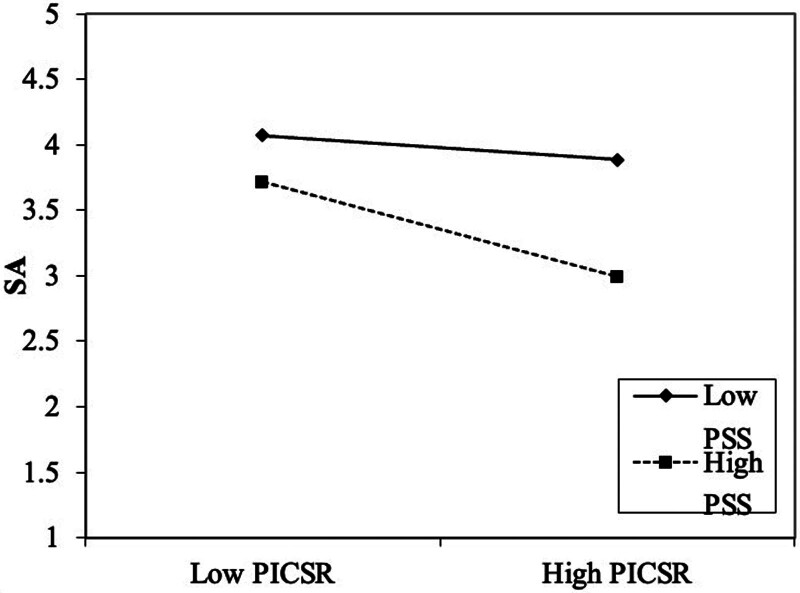
Simple slope analysis – effects of PICSR and PSS on SA. This figure illustrates the interaction between perceived internal CSR (PICSR) and perceived social support (PSS) on surface acting (SA). The X-axis represents the levels of PICSR (low to high), and the Y-axis shows the level of SA. The solid line represents the effect of low PSS, while the dashed line represents the effect of high PSS. The results indicate that the relationship between PICSR and SA is more potent at higher levels of PSS, as shown by the steeper slope of the dashed line (high PSS).

As shown in Table 8, at a high level of the moderating variable PSS, the regression coefficient of PICSR on SA is −0.363, with a significance level of 0.000, <0.05. This indicates that at a high level of PSS, PICSR has a significant negative effect on SA. At a low level of PSS, the regression coefficient of PICSR on SA is −0.090, with a significance level of 0.001, which is also <0.05. This suggests that PICSR still exerts a significant adverse effect on SA at a low level of PSS. Furthermore, the negative impact of PICSR on SA is more substantial when PSS is at a high level (see Fig. 4).

##### 4.3.3.2. Simple slope analysis of the moderating effect of PSS on the relationship between PICSR and DA

We present the results of the simple slope analysis for the independent variable (PICSR) and the moderator (PSS) on the dependent variable (DA) in Table 9.

**Table 9:** Regression coefficients of PICSR on DA at different PSS levels.

Moderator level	Regression coefficient	Standard error	*t*	*P*	95% CI	
Mean	0.144	0.030	4.867	.000	0.086	0.202
High level (+1 SD)	0.260	0.042	6.226	.000	0.178	0.342
Low level (-1 SD)	0.027	0.029	0.938	.349	-0.029	0.083

DA = deep acting, PICSR = perceived internal corporate social responsibility, PSS = perceived social support.

**Figure 5.**
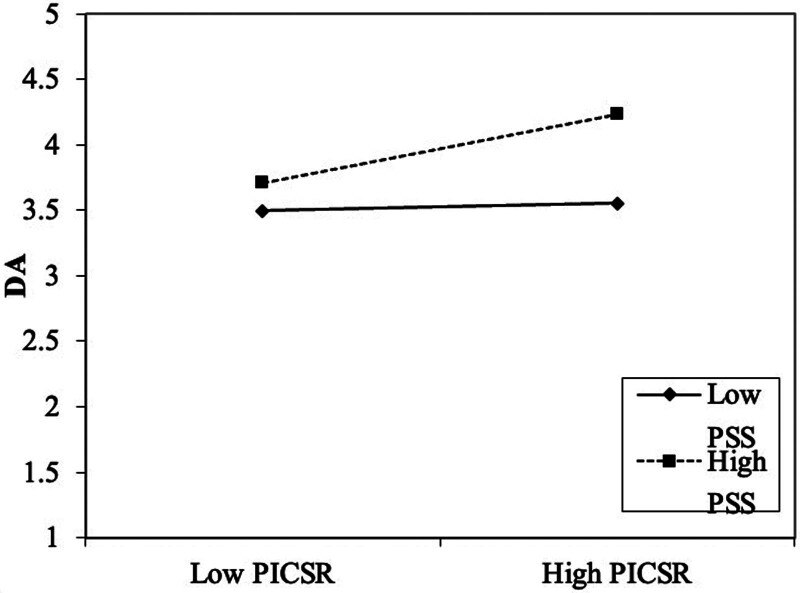
Simple slope analysis – effects of PICSR and PSS on DA. This figure shows the interaction between perceived internal CSR (PICSR) and perceived social support (PSS) on deep acting (DA). The X-axis represents the levels of PICSR (low to high), and the Y-axis shows the level of DA. The solid line represents the effect of low PSS, while the dashed line represents the effect of high PSS. The results indicate that the relationship between PICSR and DA is more potent at higher levels of PSS, as reflected by the steeper slope of the dashed line (high PSS).

As shown in the table, at a high level of PSS, the regression coefficient of PICSR on DA is 0.260, with a significance of 0.000 (*P* < .05), indicating a significant positive effect. At a low level of PSS, the regression coefficient is 0.027, with a significance of 0.349 (*P* > .05), suggesting that the effect is insignificant. These results indicate that the positive impact of PICSR on DA is more substantial when PSS is at a high level (see Fig. 5).

##### 4.3.3.3. Simple slope analysis of the moderating effect of PSS on the relationship between PECSR and DA

The results of the simple slope analysis for the independent variable (PECSR) and the moderator (PSS) on the dependent variable (DA) are presented in Table 10.

**Table 10:** Regression coefficients of PECSR on DA at different PSS levels.

Moderator level	Regression coefficient	Standard error	*t*	*P*	95% CI	
Mean	0.314	0.039	7.957	.000	0.236	0.391
High level (+1 SD)	0.603	0.054	11.235	.000	0.498	0.708
Low level (-1 SD)	0.025	0.042	0.589	.556	-0.057	0.106

DA = deep acting, PECSR = perceived external corporate social responsibility, PSS = perceived social support.

**Figure 6.**
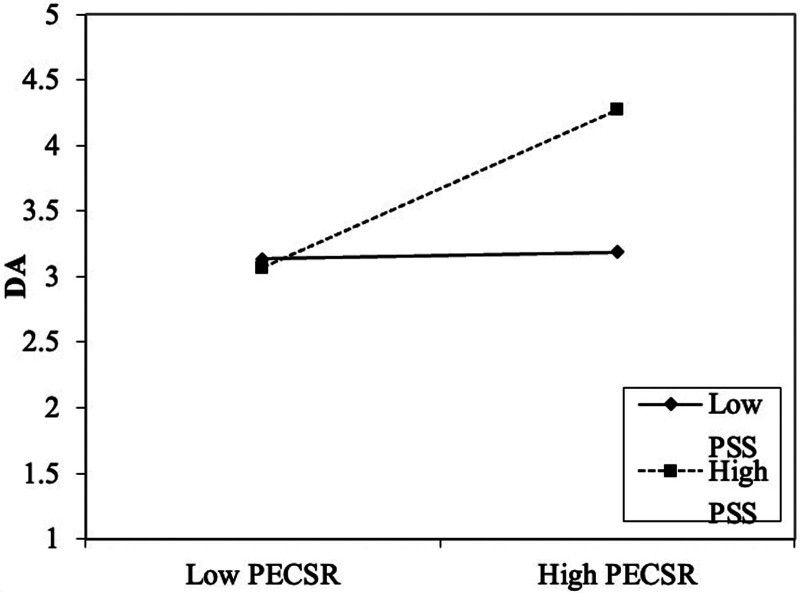
Simple slope analysis – effects of PECSR and PSS on DA. This figure illustrates the interaction between perceived external CSR (PECSR) and perceived social support (PSS) on deep acting (DA). The X-axis represents low and high levels of PECSR, and the Y-axis shows the level of DA. The solid line represents the effect of low PSS, while the dashed line represents the effect of high PSS. The results show that the relationship between PECSR and DA is more potent at higher levels of PSS, as indicated by the steeper slope of the dashed line (high PSS).

As shown in the table, at a high level of PSS, the regression coefficient of PECSR on DA is 0.603, with a significance of 0.000 (*P* < .05), indicating a significant positive effect. At a low level of PSS, the regression coefficient is 0.025, with a significance of 0.556 (*P* > .05), suggesting that the effect is insignificant. These results indicate that the positive impact of PECSR on DA is more substantial when PSS is at a high level (see Fig. 6).

These findings reinforce the moderating role of PSS, suggesting that PSS influences the extent to which perceived CSR impacts emotional labor strategies.

## 5. Discussions

This study examined how perceived corporate social responsibility affects the emotional labor of clinicians in Chinese Grade 3A public hospitals. The study’s findings revealed that PICSR substantially affected both dimensions of EL, negatively correlating with SA and positively correlating with DA (consistent with Hypotheses 1 and 2). This suggests that when clinicians perceive the organization’s efforts in internal CSR (such as employee welfare and career development), they are more likely to reduce SA and more inclined to align their inner feelings with the emotional expectations of their professional roles, thereby increasing DA. This finding aligns with previous research,^[7]^ highlighting the significant role of internal CSR in promoting employees’ adoption of positive emotional regulation strategies. However, this is inconsistent with some of the findings of Khan, who used frontline bank employees as the subject of the study and found that the effect of perceived internal and external CSR on both SA and DA was not verified.^[43]^ This may be because clinicians, as a group with naturally higher social responsibility, have significantly different emotional labor characteristics and perceived corporate social responsibility compared with other service industries. The finding also suggests that, compared to PECSR, PICSR practice activities in hospitals significantly impact clinicians more. This supports the finding of Aggarwal and Singh, which indicates that employees respond more positively to CSR strategies that focus on self-interest and directly address their personal needs rather than external CSR strategies.^[62]^ A possible reason for this is that the primary respondents in this study were female, married, and aged between 36 and 45, a group that faces significant stress and challenges in balancing work, family, and personal development. They need more accurate and direct help from the organization, such as flexible working hours, convenient childcare services, fair pay and benefits, and recognition of the unique contributions of female clinicians, which are the scope of PICSR practice activities.

However, the study’s findings revealed that PECSR did not significantly affect the 2 dimensions of emotional labor (SA and DA), meaning that Hypotheses 3 and 4 were not supported. This finding aligns with Khan et al,^[43]^ who suggested that internal CSR directly impacts employee behaviors. Moreover, it is consistent with Zhao, Wu, et al,^[5]^ who argued that external CSR tends to have a more relative or indirect influence. Clinicians focus on patient conditions and treatment outcomes in their daily work, which may lead them to perceive external CSR initiatives less strongly than other employees. When they perceive the hospital’s external CSR activities as irrelevant or secondary to their interests, it may affect their willingness to engage in CSR initiatives.^[63]^ The effectiveness of PECSR could also be affected by the organization’s internal culture. If the internal culture of the hospital is not aligned with external CSR initiatives, clinicians may develop skepticism or indifference toward these activities, reducing their influence on emotional labor behaviors. The findings of this study offer crucial insights: in designing and implementing external CSR initiatives, hospital managers should explore ways to better align them with clinicians’ personal needs and core values. This includes enhancing doctors’ sense of participation in activities that target community, environmental, and consumer interests to increase their OI.

The study’s findings also revealed that OI significantly mediated between the 2 dimensions of PCSR (PICSR and PECSR) and the 2 dimensions of EL (SA and DA), meaning that Hypotheses 5 to 12 were supported. This suggests that OI is a critical bridge between PCSR and EL. Internal and external CSR enhances OI and is associated with reduced SA (i.e., adjusting outward expressions without changing internal emotions) and increased DA among clinicians. These findings are consistent with previous research, emphasizing that employees’ perception of their organization’s social responsibility behavior, whether towards internal employees or society and the environment, enhances their sense of OI.^[36,37]^ These findings are consistent with previous research, indicating that stronger OI enhances employees’ internalization of the organization’s values and goals.^[45,64]^ This internalization fosters greater consistency between genuine and expected organizational emotions.^[65,66]^ Such consistency encourages employees to cope with emotional demands by adjusting their internal emotions rather than merely modifying outward expressions. As a result, they are less likely to engage in SA and more frequently display DA, which aligns with organizational expectations.^[28,67]^

In addition, PSS moderated the relationship between PICSR and both SA and DA (supporting H13 and H14) and between PECSR and DA (supporting H16). However, it did not significantly moderate the relationship between PECSR and SA (H15 was not supported). This finding is consistent with previous research.^[68,69]^ Specifically, social support acts as a regulatory mechanism with a buffering effect, suggesting that when clinicians feel supported by family, supervisors, and colleagues, they are more capable and willing to display positive emotions at work; this support can reduce the pressure associated with internal CSR and help them establish self-worth, reducing the likelihood of SA and increasing the expression of DA.^[49]^ When clinicians perceive high levels of social support, they are more likely to positively view external CSR initiatives, strengthening their sense of mission and belonging and promoting genuine emotional expression through DA.^[43,70]^ However, PSS does not significantly moderate the relationship between PECSR and SA. External CSR initiatives may be considered irrelevant to doctors’ daily tasks, and external pressures, such as patient demands and organizational expectations, can also overshadow the effect of social support on SA.

Lastly, these findings have theoretical and practical significance. Theoretically, this study examines the link between perceived CSR and emotional labor from a micro-perspective, expanding the research field of CSR and offering new perspectives and empirical support to address the inconsistencies in existing research. From the standpoint of buffering theory, this study introduces PSS as a moderating variable between PCSR and EL, further improving the related research. In addition, this study offers practical implications for hospital administrators, providing specific CSR practices that can directly benefit clinicians. For instance, creating academic support platforms effectively reduces clinicians’ dual pressures between research and clinical practice, helping them better balance both activities and alleviating the emotional labor associated with divided attention. Expanding auxiliary teams to ease the non-clinical workload of clinicians and setting up multimedia support departments to assist in case documentation and academic report preparation, thus enhancing research and teaching efficiency.

Furthermore, providing comprehensive welfare programs, including health insurance, legal support, and retirement planning, while implementing mental health and stress management initiatives to reduce the emotional labor burden on clinicians. A dedicated patient feedback office can promptly address patient concerns, reducing the communication burden on clinicians and preventing emotional exhaustion from patient reactions. Strengthening team collaboration support can ease individual doctors’ workloads, lessening emotional labor intensity. Finally, providing flexible work arrangements, leave policies, and family support options such as childcare leave will help clinicians achieve a better work-life balance, further alleviating the emotional labor associated with their professional responsibilities.

## 6. Conclusions

This study explores the impact of CSR on emotional labor from a micro-perspective of perceived CSR, addressing a research gap by examining CSR as an organizational-level factor influencing individual emotional labor. Drawing on Social Identity Theory and Buffering Theory, it provides a theoretical explanation of the underlying mechanisms, thereby broadening the scope of emotional labor research. The findings reveal that internal CSR exerts a more positive influence on clinical physicians’ emotional labor, highlighting the importance of hospital administrators ensuring that external CSR initiatives are directly relevant to physicians’ interests. Furthermore, by incorporating PSS as a moderating variable, this study offers new empirical evidence demonstrating that social support effectively buffers the relationship between perceived internal CSR and SA and DA while also playing a partial buffering role in the relationship between perceived external CSR and DA.

However, this study has several limitations. First, this study relied primarily on self-reported data from clinicians. Although this method applies to variables related to self-perception, it may introduce bias or inaccuracies. Second, our study only examined the emotional labor of clinicians in the Chinese cultural context, and whether the findings apply to countries with different cultural backgrounds remains to be verified. There may be sample selection bias because we may have included only specific types or regions of clinicians, which could constrain the generalizability of the findings. Third, this study only discusses the mediating role of OI and the moderating role of PSS in the effect of perceived corporate social responsibility on emotional labor, and other moderating pathways may be worth exploring.

Researchers can make several recommendations for future research to address the shortcomings of this study. Future research should consider employing longitudinal study designs to track the dynamic trajectories of emotional labor among clinicians, examining fluctuations across daily, weekly, and yearly timeframes. Additionally, exploring how career stages, age, and key professional events (e.g., promotions, role transitions, significant medical incidents) shape these patterns over time is crucial. Furthermore, with the advancement of telemedicine and digital healthcare, the role of CSR in virtual medical environments remains an area requiring more profound investigation. For instance, different communication modalities (e.g., video, audio, text) may influence clinicians’ emotional expression and perceptions of social support, potentially altering the impact of CSR on emotional labor. Understanding how CSR can help healthcare professionals navigate these evolving work environments is essential for designing sustainable workplace policies. Finally, the cultural variations in the relationship between CSR and emotional labor warrant further exploration. In highly collectivist cultures (e.g., China) versus highly individualistic cultures (e.g., Western countries), CSR may influence clinicians’ OI and emotional regulation through distinct psychological mechanisms. Future research could benefit from cross-cultural comparative studies incorporating multi-national samples to examine the effectiveness of CSR across different healthcare systems and identify potential cultural moderators, thereby offering more targeted theoretical and practical insights.

## Acknowledgments

The authors sincerely thank all the participants for their valuable contributions to our survey, which provided essential data for this study. Special thanks to Hongmin Jia for his kind assistance with data collection, which was instrumental to our research.

## Author contributions

**Conceptualization:** Mohd Anuar Arshad.

**Data curation:** Mengjiao Zhao.

**Investigation:** Guoyu Luo.

**Methodology:** Guoyu Luo.

**Software:** Mengjiao Zhao.

**Supervision:** Mohd Anuar Arshad.

**Validation:** Mohd Anuar Arshad.

**Writing – original draft:** Guoyu Luo.

**Writing – review & editing:** Xianhang Xu.
